# Cost-effective and robust genotyping using double-mismatch allele-specific quantitative PCR

**DOI:** 10.1038/s41598-019-38581-z

**Published:** 2019-02-15

**Authors:** Steve Lefever, Ali Rihani, Joni Van der Meulen, Filip Pattyn, Tom Van Maerken, Jo Van Dorpe, Jan Hellemans, Jo Vandesompele

**Affiliations:** 1 Center for Medical Genetics Ghent, Ghent University, Ghent, 9000 Belgium; 2 Biogazelle, Zwijnaarde, 9052 Belgium; 3 Cancer Research Institute Ghent (CRIG), Ghent University, Ghent, 9000 Belgium; 4 Bioinformatics Institute Ghent (BIG), Ghent University, Ghent, 9000 Belgium; 5 Department of Pathology, University Hospital Ghent, Ghent, 9000 Belgium; 6 Present Address: Karolinska Institute, Stockholm, SE-171 77 Sweden; 7 Present Address: Ontoforce, Ghent, 9000 Belgium

## Abstract

For a wide range of diseases, SNPs in the genome are the underlying mechanism of dysfunction. Therefore, targeted detection of these variations is of high importance for early diagnosis and (familial) screenings. While allele-specific PCR has been around for many years, its adoption for SNP genotyping or somatic mutation detection has been hampered by its low discriminating power and high costs. To tackle this, we developed a cost-effective qPCR based method, able to detect SNPs in a robust and specific manner. This study describes how to combine the basic principles of allele-specific PCR (the combination of a wild type and variant primer) with the straightforward readout of DNA-binding dye based qPCR technology. To enhance the robustness and discriminating power, an artificial mismatch in the allele-specific primer was introduced. The resulting method, called double-mismatch allele-specific qPCR (DMAS-qPCR), was successfully validated using 12 SNPs and 15 clinically relevant somatic mutations on 48 cancer cell lines. It is easy to use, does not require labeled probes and is characterized by high analytical sensitivity and specificity. DMAS-qPCR comes with a complimentary online assay design tool, available for the whole scientific community, enabling researchers to design custom assays and implement those as a diagnostic test.

## Introduction

Various methods are available for single nucleotide polymorphism (SNP) genotyping, each with their benefits and limitations. While SNP genotyping on a genome-scale is currently dominated by microarray analysis or massively parallel sequencing, assessment of a targeted selection of SNPs (or single nucleotide variants) is mainly done through PCR. Restriction fragment length polymorphism (RFLP)^1^ is possibly the oldest and simplest method of this type relying on restriction digest followed by size separation of the differential fragments. PCR based methods, including high-resolution melting (HRM)^2,3^ and real-time PCR using hydrolysis probes^4^ are faster and less laborious. Although these PCR based methods can be performed in high-throughput, are amenable to automation, and generate data that is relatively easy to analyze, a major drawback is the dependency on probes – either labeled or not – increasing design complexity and cost, especially when the number of samples to be genotyped is low. As an alternative, we present a cost-effective quantitative PCR (qPCR) based genotyping assay consisting of two parallel qPCR reactions, each including a modified allele-specific (AS) forward and common wild-type (WT) reverse primer (or vice versa). The combined information of quantification cycle values (Cq) from the two allele-specific reactions enables robust and straightforward genotyping. The simple setup avoids the necessity for probes, dedicated software and specialized instrumentation (and accompanying software) such as a high-resolution melting or sequencing instrument. Our method gives accurate genotyping results with all types of SNPs across a wide range of DNA input concentrations. Allele-specific qPCR assay design is available through our in-house developed primerXL web tool (www.primerxl.org) to increase adoption throughout the community.

## Methods

### Primer and template design

**Figure 1:**
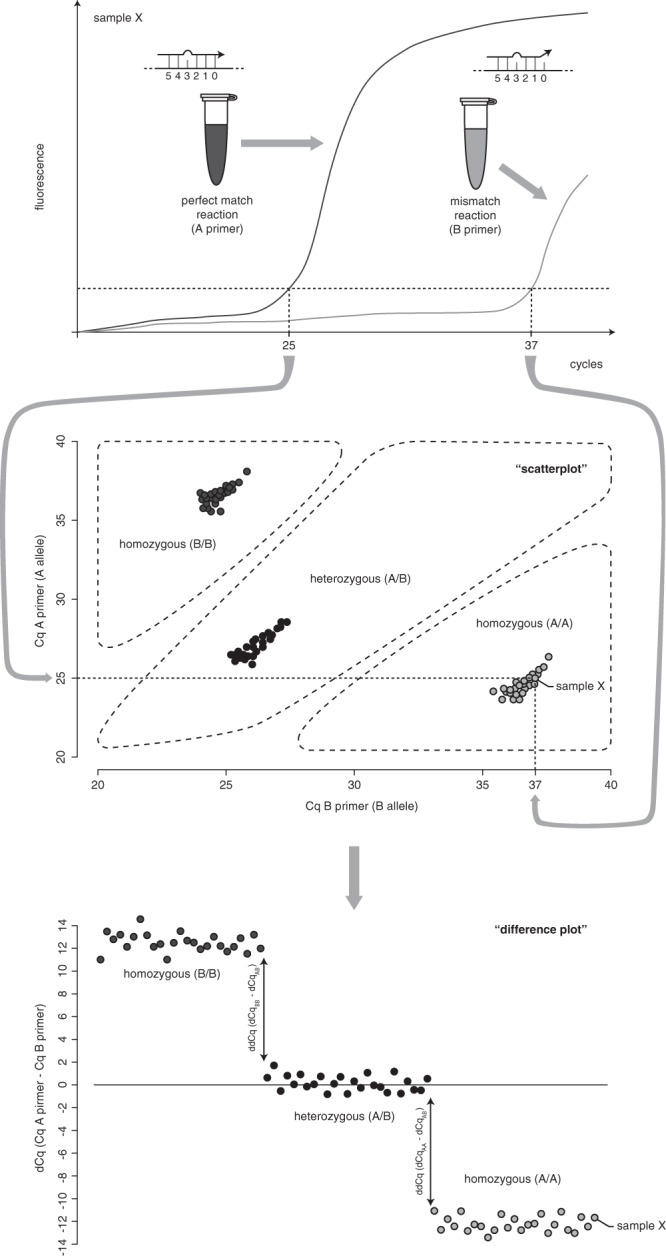
Schematic representation of DMAS-qPCR results for one assay on one representative homozygous AA sample (sample X - top panel, qPCR amplification curves) and 75 samples (one third homozygous AA, heterozygous AB and homozygous BB) as scatter (middle) and difference plots (bottom).

SNP (or mutation) specific qPCR assays were designed using our in-house developed primerXL web tool (www.primerxl.org). An assay consists of one common reverse primer and two or more allele-specific (AS) forward primers with their 3′ end (position 0, Fig. 1) overlapping the SNP. Unless stated otherwise, an additional mismatch at position 3 (i.e. the fourth nucleotide from the primer 3′ end) was introduced to create double-mismatch AS primers (DMAS primers) and increase genotype discrimination power. By definition, each DMAS primer is referred to as a ‘match’ for the corresponding allele, and a ‘mismatch’ for the alternative allele. A custom Primer3^5^ and UNAfold^6^ based perl script was used to generate 60 bp synthetic templates corresponding to the alleles and to be used as positive control templates. If possible, templates were kept free of secondary structures.

### Cell cultures

Cell lines where cultured in RPMI medium, enriched with 10% fetal calf serum (FCS). All cell lines are regularly genotyped to confirm their identity and tested for mycoplasma contamination. The cell lines were obtained between 1993 and 2010 from Peter Ambros (STA-NB-12, STA-NB-3, STA-NB-8, STA-NB-9, and STA-NB-10), Garrett Brodeur (NGP, NLF, and NMB), Susan Cohn (NBL-S, SHEP), Valérie Combaret (CLB-GA), Thomas Look (SJNB-1, SJNB-8, and SJNB-10), John Lunec (SK-N-BE, SK-N-BE(2c)), Sven Påhlman (SH-SY5Y), Patrick Reynolds (CHP-134, CHP-901, CHP-902R, SMS-KAN, and SMS-KCNR), and Rogier Versteeg (GICIN-1, IMR-32, LA-N-1, LA-N-2, LA-N-5, LA-N-6, N-206, SK-N-AS, SK-N-FI, SK-N-SH, and TR-14), or established in our laboratory (UHG-NP).

### Single mismatch reactions

AS assays (without additional mismatch) were designed for 6 SNPs involved in the *TP53* pathway. qPCR was performed in duplicate on a LightCycler480 (Roche) instrument. For assay validation, two homozygous (one for each allele) and one heterozygous (1:1 mix of the two templates) synthetic sample consisting of 10 000 template molecules were assessed. For all synthetic samples, a 6-point 10-fold standard curve using perfect matching forward and reverse primers was run. All 5 µl qPCR reactions were performed using the Bio-Rad SsoAdvanced SYBR Green Supermix, according to the manufacturer’s recommendations with a final primer concentration of 0.25 µM and 2 µL template input (2 min at 95 °C; 44 cycles of 5 s at 95 °C and 30 s at 60 °C).

### Determination of optimal artificial mismatch position

AS assays harboring all possible nucleotides at positions 1 to 5 were designed for 6 SNPs involved in the *TP53* pathway (Table 1). qPCR was performed in duplicate on a LightCycler480 (Roche) instrument. For assay validation, two homozygous (one for each allele) and one heterozygous (1:1 mix of the two templates) synthetic sample consisting of 10 000 template molecules were assessed. For all synthetic samples, a 6-point 10-fold standard curve using perfect matching forward and reverse primers was run. All 5 µl qPCR reactions were performed using the Bio-Rad SsoAdvanced SYBR Green Supermix, according to the manufacturer’s recommendations with a final primer concentration of 0.25 µM and 2 µL template input (2 min at 95 °C; 44 cycles of 5 s at 95 °C and 30 s at 60 °C).

**Table 1:** SNPs and their corresponding oligonucleotide sequences.

	allele	forward	reverse	template
rs6734469	A/G	TCCCCCACTGATTTCTTCTGTAC[**A/C/G/T**]	ACTGTCAGCACCAGAGGGAC	TCCCCCACTGATTTCTTCTGTAC[**A/G**]AGATACTCTACTCTGGGTCCCTCTGGTGCTGACAGT
rs187115	C/T	CAGCGAGATAGTTGTGCTGACA	GAGTGAGAAAGATGTTATCATCGTCATTG[**A/C/G/T**]	CAGCGAGATAGTTGTGCTGACAGTCATATA[**C/T**]CAATGACGATGATAACATCTTTCTCACTC
rs1027154	C/G	CTCCATACACTTTCTTAGGTGACTTTT[**A/C/G/T**]	ACCAGAGCATTCACCCTCAG	CTCCATACACTTTCTTAGGTGACTTTT[**C/G**]CGAAGTGAACCACTGAGGGTGAATGCTCTGGT
rs2168945	A/C	GGAATAGGAAATGTGATGCAAGCAGA[**A/C/G/T**]	TTGAATAGGGCAACGACAGATGA	GGAATAGGAAATGTGATGCAAGCAGA[**A/C**]CAGGTTTACGTCATCTGTCGTTGCCCTATTCAA
rs4245739	A/C	GAGAAAAGACCACGAGACGG	TTTTCAAATAATGTGGTAAGTGAAC[**A/C/G/T**]	GAGAAAAGACCACGAGACGGAGACTGATTTGGCC[**A/C**]GTTCACTTACCACATTATTTGAAAA
rs2273953	A/G	TTCCTTCCTTCCTGCAGAGC[**A/C/G/T**]	AGAGCTCCAGAGGTGCTCAAA	TTCCTTCCTTCCTGCAGAGC[**A/G**]AAGAATTCACTGTGACTATTTGAGCACCTCTGGAGCTCT

Mismatches and allele-specific nucleotides are marked bold and underlined.

### Effect of input concentration

DMAS-qPCR assays were designed for the SNPs used in the single mismatch experiment, and 6 additional SNPs involved in the same pathway (Table 2). A 4-point, 4-fold dilution series (16, 4, 1 and 0.25 ng was created for eight tumor cell lines (SJNB-8, CHP-901, SHEP, SJNB-6, GIMEN, CHP-134, N-206 and SH-SY5Y). PCR reactions were performed as described earlier on a LightCycler480 (Roche) instrument: two parallel qPCR reactions, each with a different allele-specific forward primer and a common reverse primer.

**Table 2:** SNPs and their corresponding oligonucleotide sequences.

	allele	forward	reverse	template
rs6734469	A/G	TCCCCCACTGATTTCTTCTG**C**AC[**A/G**]	ACTGTCAGCACCAGAGGGAC	TCCCCCACTGATTTCTTCTGTAC[**A/G**]AGATACTCTACTCTGGGTCCCTCTGGTGCTGACAGT
rs187115	C/T	CAGCGAGATAGTTGTGCTGACA	GAGTGAGAAAGATGTTATCATCGTCA**C**TG[**A/G**]	CAGCGAGATAGTTGTGCTGACAGTCATATA[**C/T**]CAATGACGATGATAACATCTTTCTCACTC
rs1027154	C/G	CTCCATACACTTTCTTAGGTGACT**C**TT[**C/G**]	ACCAGAGCATTCACCCTCAG	CTCCATACACTTTCTTAGGTGACTTTT[**C/G**]CGAAGTGAACCACTGAGGGTGAATGCTCTGGT
rs2168945	A/C	GGAATAGGAAATGTGATGCAAGC**C**GA[**A/C**]	TTGAATAGGGCAACGACAGATGA	GGAATAGGAAATGTGATGCAAGCAGA[**A/C**]CAGGTTTACGTCATCTGTCGTTGCCCTATTCAA
rs4245739	A/C	GAGAAAAGACCACGAGACGG	TTTTCAAATAATGTGGTAAGTG**C**AC[**G/T**]	GAGAAAAGACCACGAGACGGAGACTGATTTGGCC[**A/C**]GTTCACTTACCACATTATTTGAAAA
rs2273953	A/G	TTCCTTCCTTCCTGCAG**C**GC[**A/G**]	AGAGCTCCAGAGGTGCTCAAA	TTCCTTCCTTCCTGCAGAGC[**A/G**]AAGAATTCACTGTGACTATTTGAGCACCTCTGGAGCTCT
rs319227	G/T	CCCTGCTCTAGAACATCGAC**C**TA[**A/C**]	GCAGACCCAGGACTTGAATGC	CCCTGCTCTAGAACATCGACATA[**A/C**]GTGTACCCTGTATTGGCATTCAAGTCCTGGGTCTGC
rs2069347	C/T	CATATTTTAGTCCTCGGTATCTAACA**A**AG[**T/C**]	ATGACTTGTACATAAGAGCAACGATCT	CATATTTTAGTCCTCGGTATCTAACACAG[**C/T**]CTGAGATCGTTGCTCTTATGTACAAGTCAT
rs2426127	C/T	GGCTGACAGAATTCCTTTTTAGATGC	CCAAAGACCCTTAGCCCTAA**A**TC[**A/G**]	GGCTGACAGAATTCCTTTTTAGATGCGAGTGCTCCG[**C/T**]GAGTTAGGGCTAAGGGTCTTTGG
rs1800054	C/G	ATTAAACATCTAGATCGGCATTCAG**C**TT[**C/G**]	TGCAAGGCATAATGATATATAGGAAGCAA	ATTAAACATCTAGATCGGCATTCAGATT[**C/G**]CATTGCTTCCTATATATCATTATGCCTTGCA
rs34330	C/T	CGTCGGGGTCTGTGTCTTTTG	AACAAAGCGCCCCTA**A**GC[**A/G**]	CGTCGGGGTCTGTGTCTTTTGCGGGGGGAGGGATCGAAATA[**C/T**]GCGTAGGGGCGCTTTGTT
rs1045485	C/G	TGCTCTCCAGCTGTGGTCTG	AGATTTGCTCTACTGTGCAGT**A**AT[**C/G**]	TGCTCTCCAGCTGTGGTCTGCTACAACTGGTAGGT[**C/G**]ATGACTGCACAGTAGAGCAAATCT

Mismatches and allele-specific nucleotides are marked bold and underlined. The first 6 SNP assays were used for the determination of the optimal artificial mismatch location, assessment of the required DNA input concentration and assay specificity. The latter 6 SNP assays were only applied in the assay specificity and DNA input concentration experiments.

### Assessment of relative analytical sensitivity

DMAS-qPCR assays were designed for 15 clinically relevant mutations (Table 3). For each of the 48 selected tumor cell lines (ACN, CHLA-90, CHP-134, CHP-901, CHP-902R, CLB-GA, GICIN-1, GIMEN, IMR-32, Kelly, LA-N-1, LA-N-6, LA-N-6 (T10-295), LA-N-2, LA-N-5, N-206, NB-1, NB-13, NB-5, NBL-S, NGP, NLF, NMB, SH-SY5Y, SH-EP, SJNB-10, SJNB-12, SJNB-6, SJNB-8, SJNB-1, SK-N-AS, SK-NF-I, SK-MYC2, SK-NB-E, SK-N-BE(2c), SKNSH, SMS-KAN, SMS-KCN, SMS-KCNR, STA-NB-10, STA-NB-12, STA-NB-3, STA-NB-8, STA-NB-9, TR-14, UHG-NP and UKF-NB3), 4 ng DNA input was subjected to each of the DMAS-qPCR assays with a final primer concentration of 0.25 µM per 5 µL reaction. In addition, the sensitivity of these assays was tested using synthetic templates. For this, a 14-point, 2-fold mutation in wild-type – and vice versa – dilution series was created (mutation/WT: 50/50, 25/75, 12.5/87.5, 6.25/93.75, 3.13/96.87, 1.56/98.44, 0.78/99.22, 0.39/99.61, 0.20/99.80, 0.10/99.90, 0.05/99.95, 0.025/99.975, 0.013/99.987, 0/100), followed by qPCR on a LightCycler 480 (Roche) instrument using a total of 100 000 input molecules per 5 µL reaction and a final primer concentration of 0.25 µM.

**Table 3:** Clinically relevant mutations and their corresponding oligonucleotide sequences.

		allele	allele-specific primers	common primer
rs113488022	BRAF (V600E)	A/T	AATAGGTGATTTTGGTCTAGCTA**C**AG[**A/T**]	GTAACTCAGCAGCATCTCAGGG
rs113993960	CF (deltaF508)	C/T	GCCTGGCACCATTAAAGAAAATAT**C**AT[**C/T**]	GGCATGCTTTGATGACGCTTC
rs6025	Leiden mutation (FCTRV)	C/T	ACTTCAAGGACAAAATACCTGTATT**C**CT[**C/T**]	TCGCCTCTGGGCTAATAGGAC
rs77375493	JAK2 (V617F)	A/C	AGTTTTACTTACTCTCGTCTCCACAGA[**A/C**]	AGCAGCAAGTATGATGAGCAAGC
COSM28057	ALK (F1174L_A > G)	C/T	CCTCTCTGCTCTGCAGC**A**AA[**C/T**]	GGGTCTCTCGGAGGAAGGAC
COSM28059	ALK (F1174C)	A/G	CCTCTCTGCTCTGCAGCA**A**AT[**G/T**]	GGGTCTCTCGGAGGAAGGAC
COSM28055	ALK (F1174L_G > T)	A/C	CTCTCTGCTCTGCAGCAA**A**TT[**A/C**]	GGGTCTCTCGGAGGAAGGAC
COSM28056	ALK (R1275Q)	C/T	CAGTCTTTACTCACCTGTAGATG**T**CT[**C/T**]	GCCAGAAACTGCCTCTTGACC
rs17851045	KRAS (codon 61)	A/T	CCCTCATTGCACTGTACTC**C**TC[**A/T**]	TTGTCCGTCATCTTTGGAGCAG
COSM12429	EGFR (L858R)	G/T	TGTCAAGATCACAGATTTTG**G**GC[**G/T**]	CTAGTGGGAAGGCAGCCTGG
COSM6252	EGFR (G719S)	A/G	ACTGAATTCAAAAAGATCAAAGTG**C**TG[**A/G**]	AGACCATGAGAGGCCCTGC
COSM6253	EGFR (G719C_G > T)	G/T	ACTGAATTCAAAAAGATCAAAGTG**C**TG[**G/T**]	AGACCATGAGAGGCCCTGC
COSM18441	EGFR (G719C_GG > TT)	A/C	GAACGCACCGGA**G**CC[**A/C**]	TGGAGCCTCTTACACCCAGTG
COSM6239	EGFR (G719A)	C/G	TGAATTCAAAAAGATCAAAGTGC**T**GG[**C/G**]	GCTCCCCACCAGACCATGAG
COSM6240	EGFR (T790M)	C/T	CCACCGTGCAGCTCA**T**CA[**C/T**]	AGCAGGTACTGGGAGCCAAT

Mismatches and allele-specific nucleotides are marked bold and underlined.

### Hydrolysis probe based qPCR assays

Assays were performed using the TaqMan SNP Genotyping mastermix with 0.125 µL of the 40x SNP genotyping assay and 9.5 ng input DNA in a 5 µl reaction on a 7900HT (Applied Biosystems) instrument (Table 4). The following protocol was used: 2 min at 50 °C; 10 min at 95 °C; 40 cycles of 15 s at 95 °C and 60 s at 60 °C.

**Table 4:** SNPs and their corresponding TaqMan assay IDs.

	Reference	TaqMan assay ID
rs6734469	^10^	C__29724290_10
rs187115	^10^	C____779820_10
rs1027154	^10^	C___1935268_20
rs2168945	^10^	C___1673863_10
rs4245739	^11^	C__11623776_10
rs2273953	^12^	C__16180357_10
rs319227	^10^	C____803346_10
rs2069347	^10^	C___2000410_20
rs2426127	^10^	C__16230087_10
rs1800054	^12^	C___2283268_20
rs34330	^12^	C___2402292_10
rs1045485	^12^	C___8823877_20

### FFPE DMAS-qPCR

FFPE-DNA was extracted from tumor lung biopsies by means of the QIAamp DNA FFPE Tissue kit (Qiagen) using three to five 8–10 µM sections. DMAS-qPCR reactions for three EGFR mutations (T790M, L858R and G719A) were performed on a LightCycler 480 instrument (Roche), using 16 ng input DNA per 5 µL reaction. Variant status was assessed using targeted resequencing on an Illumina MiSeq instrument (Nextera XT library prep, 2 × 250 cycles), followed by CLCbio Genomics Workbench (Qiagen) based variant calling. For all samples and mutations, variant allele frequencies (VAF) were determined using droplet digital PCR on a QX100 droplet generator/reader (Bio-Rad) using PrimePCR ddPCR mutation assays according to the manufacturer. All experimental protocols applied on the FFPE tumor samples were approved by the ethical committee of the University Hospital Ghent (project number 2004/094) and have been carried out in accordance with relevant guidelines and regulations. For each sample, informed consent was obtained from the corresponding patient or from a parent and/or legal guardian in case the patient was under 18 years old.

## Results

### Single mismatch AS-qPCR reactions

Allele-specific assays were designed for 6 SNPs involved in the *TP53* pathway (Table 1) and tested on eight randomly selected and previously genotyped cancer cell line samples. For each sample and SNP, four parallel reactions were performed in duplicate, each with a different AS primer. In this experiment, the four possible nucleotides were used at the 3′ end of each AS primer - resulting in three single mismatch reactions and one perfect match reaction. These AS-qPCR results indicate that the discrimination power of a single 3′ terminal mismatch is not sufficient for reliable genotype calling (Suppl. Table 1). For only two SNPs (rs4245739 and rs1027154) a clear separation between the three genotypes was observed (data not shown).

### Double mismatch AS-qPCR reactions (DMAS-qPCR)

**Figure 2:**
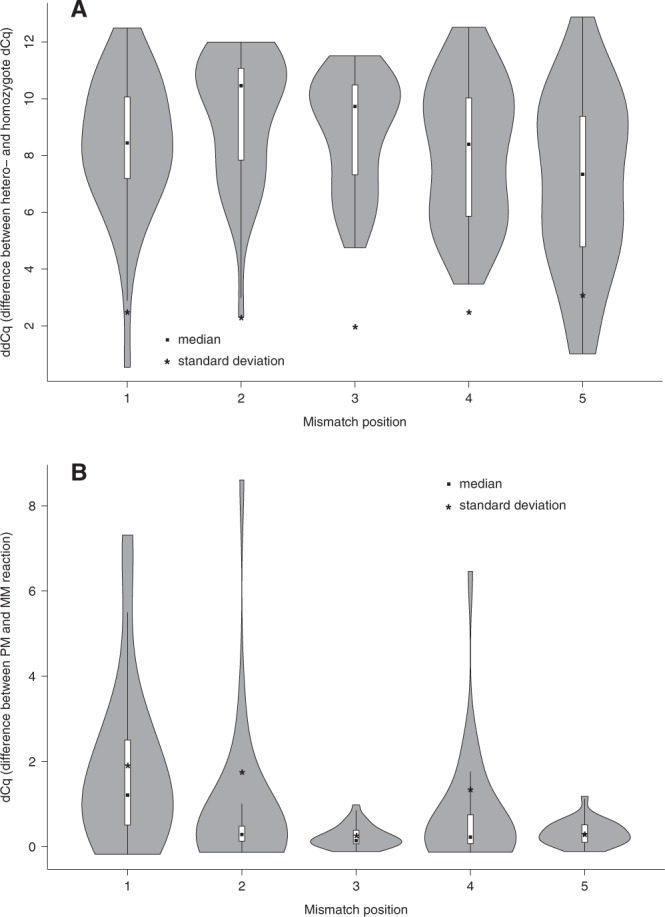
(**A**) Violin plot denoting dCq differences and standard deviation per position, between match and mismatch allele-specific primer for homozygote samples (position 0 is terminal position), (**B**) Violin plot denoting Cq differences and standard deviation, per position between ‘perfect’ match reactions – harboring no 3′ terminal mismatch – with and without artificial mismatch (position 0 is terminal position).

Based on previous work, inclusion of an artificial mismatch into the AS primer was evaluated in order to increase the genotype discrimination power^7,8^ (Table 2). This results in a double mismatch when the AS primer for allele A hybridizes to allele B (called ‘mismatch’, see Materials and Methods), and a single mismatch when primer A hybridizes to its target allele A (called ‘match’). Although the artificial mismatch may have a beneficiary effect on limiting amplification of the ‘mismatch’ reaction, the potential downside of this approach is that the artificial mismatch may negatively impact the ‘match’ reaction. To determine the optimal position of the mismatch in order to maximize the discrimination power while minimizing the effect on the ‘matching’ AS primer, all possible nucleotide changes were incorporated into positions one to five of both AS primers and tested on synthetic template samples. Data for six SNPs presented in Fig. 2 indicate that mismatches on positions two and three result in the highest average dCq differences (ddCq) (and thus highest discriminating power) between ‘match’ and ‘mismatch’ reactions (Fig. 2A). When taking into account the impact of the artificial mismatch on the match reaction (Fig. 2B) and the standard deviation of the dCq values, a mismatch on position three appears to be the optimal solution (low standard deviation, limited effect on the match and large and reproducible dCq value between ‘match’ and ‘mismatch’ reaction). Assessment of the Cq scatterplots (Suppl. Fig. 1) leads to the same conclusion with clearest clustering of the three different genotypes for an artificial mismatch on position three. These plots also clearly show the expected Cq difference of one cycle between homozygote and heterozygote genotypes, underscoring the accuracy and quantitative power of the assays. In addition to the position of the artificial mismatch, the mismatch type may in principle also affect the resolution of the genotyping assays. Although further experiments may be needed, our results suggest that there are no differential effects when comparing different base pair mismatches on position three.

### Effect of DNA input concentration

**Figure 3:**
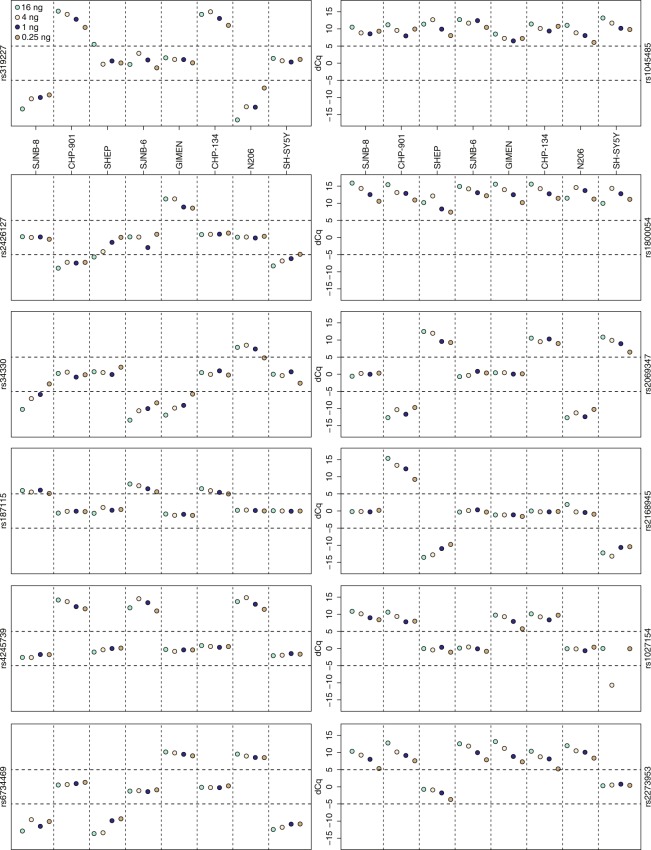
Cq difference plots – one dimensional plots of the Cq difference between the perfect and mismatch reactions of the same assay – for 12 *TP53* related SNPs in 8 neuroblastoma samples using 16, 4, 1 and 0.25 ng input DNA.

Assays for six additional SNPs (Table 2), also involved in the *TP53* pathway, were designed and tested on a serial dilution series (16, 4, 1 and 0.25 ng) of DNA samples from eight neuroblastoma cell lines to evaluate the robustness of the method with regard to the input concentration. While the data from the previous experiment suggested that the type of mismatch at position 3 had no specific effect, for the 6 new assays, the type of the artificial mismatch was chosen based on reported mismatch stability ranking: G/G > G/T ≥ G/A > T/T ≥ A/A > T/C ≥ A/C ≥ C/C (ranked from most stable to least stable mismatch)^9^. A Cq difference plot of the data (Fig. 3) demonstrates that in 98% (376/384) of the DNA sample-concentration combinations the correct genotype could be determined (using a 5 dCq cut-off). Although the discrimination power of the DMAS-qPCR assays decreases with lower concentrations of input DNA, the wide concentration range (64-fold, down to 0.25 ng input DNA or ~80 copies) at which the DMAS-qPCR assays are still able to discriminate between genotypes confirms the sensitivity and robustness of the method. Only for one SNP, namely rs34330, genotype calling becomes more challenging when using the lowest input concentration point.

### Assay specificity

**Figure 4:**
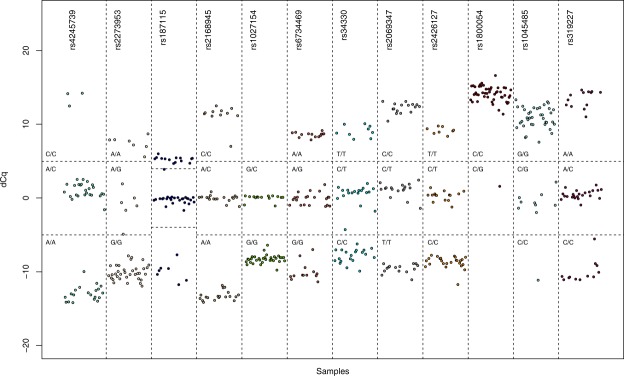
Overview of Cq difference plots for 12 *TP53* related SNPs in 48 neuroblastoma samples.

**Figure 5:**
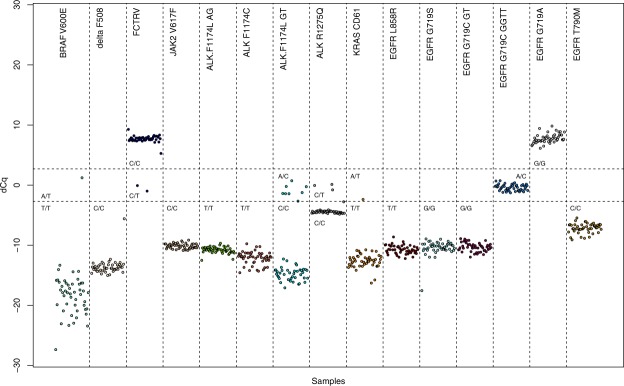
Overview of Cq difference plots for 15 clinically relevant mutations in 48 neuroblastoma samples.

To determine the accuracy of our method, the 12 DMAS-qPCR genotyping assays were tested on DNA from 48 neuroblastoma cell lines (4 ng input) for which corresponding TaqMan results were available. All of the 576 genotypes could be called using our method with a manually set threshold (100% call rate), while 23 TaqMan reactions resulted in undetermined calls (96% call rate using the TaqMan Genotyper software) (Fig. 4 and Suppl. Table 2). For the remaining 553 genotypes, results were identical for 547 genotypes when comparing DMAS-qPCR and TaqMan qPCR (98.9% concordance). When recalling the TaqMan results manually, the concordance increases to 99.5% (7 undetermined calls, 3 incorrect calls and 566 correct calls). In addition, the 48 neuroblastoma cell line cohort was subjected to DMAS-qPCR for 15 clinically relevant mutations (Fig. 5, Table 3). Genotype calling was performed identically to the *TP53* SNPs. The results show that the majority of the cell lines are negative for the respective mutations, with the exception of the *EGFR* G719C GG> TT mutation, a *BRAF* V600E variant in the ACN cell line, two FCTRV (Leiden) mutations and a few *ALK* F1174L and R1275Q mutations. Comparison of the DMAS-qPCR calls for these two latter mutations with corresponding TaqMan results available for a subset of the cell lines (n = 40) again indicate a high concordance (98.75%) (Suppl. Table 3).

### Assay sensitivity for mutation detection

**Figure 6:**
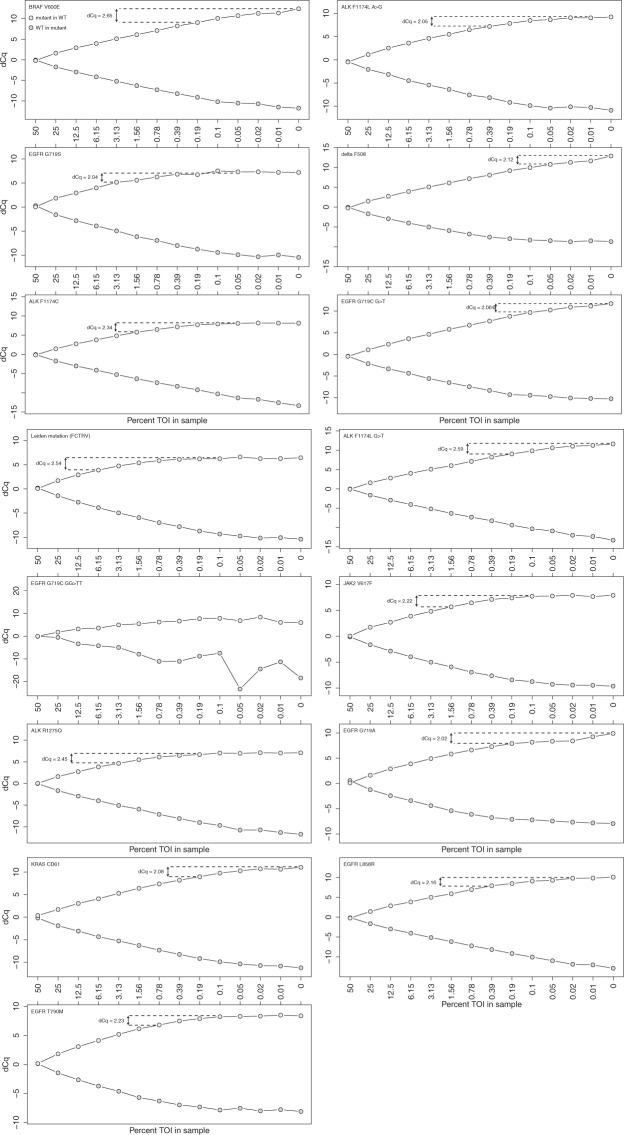
Cq difference plots for mutant in wild type (and vice versa) dilution series of the 15 clinically relevant mutations. Dotted lines display the relative sensitivity when using a conservative 2 dCq cut-off. (TOI = target of interest).

Since tumor samples are almost always contaminated with stromal cells, the sensitivity of any genotyping method has a large impact on calling accuracy. To assess the sensitivity of our DMAS-qPCR approach, synthetic templates were developed for both the mutant and wild-type allele of each of the 15 clinically relevant mutations. A 14-point, 2-fold dilution of the mutant allele in a wild-type background – and vice versa – was then created followed by DMAS-qPCR using the corresponding assay. We observe that the dCq values deviate from the theoretical trend line for the higher target dilutions (Fig. 6). This can most likely be attributed to specificity of the assay since at very low mutant concentrations, the mutant primer will start competing with the wild-type primer to amplify the wild-type allele. Nevertheless, when using a conservative 2 dCq difference to discriminate between wild-type and a mutant in wild-type sample, sensitivity scores ranging from 6.25% down to 0.05% (mutant in WT) can be reached without any optimization; nine of the 15 assays show detection sensitivities below 1%.

### Performance on FFPE tumor samples

**Figure 7:**
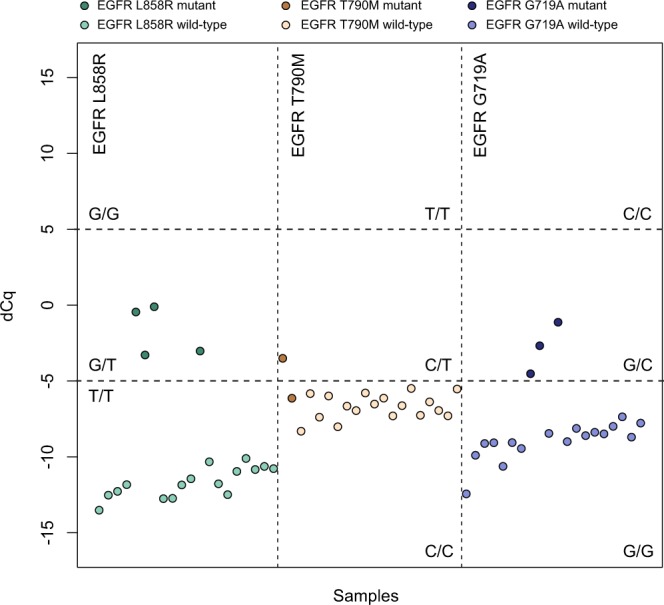
Overview of Cq difference plots for 3 *EGFR* mutations in 20 FFPE lung tumor samples.

**Figure 8:**
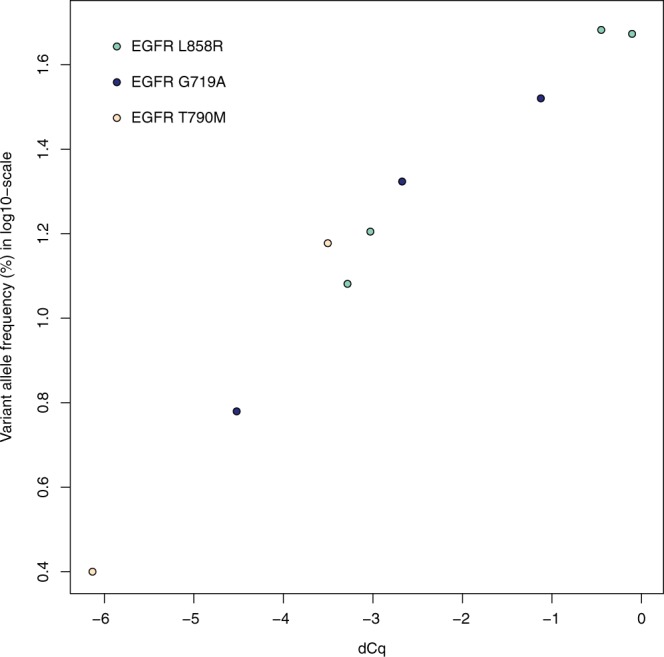
ddPCR based variant allele frequency in function of the dCq – determined by DMAS-qPCR – for each of the mutant lung tumor samples.

Since DMAS-qPCR performance can differ significantly between a setup using cell lines (or synthetic templates) and real tumor samples – due to the more heterogeneous nature of the latter – the robustness of the technique was assessed on DNA isolated from FFPE lung tumor tissues with known *EGFR* T790M, L858R and G719A mutation status. Results show that all four *EGFR* L858R mutant and all three G719A mutant samples can easily be called using a 5 dCq cut-off, with the largest discriminating power (i.e. largest dCq values for homozygous wild-type samples) being observed for the L858R assay. For the T790M mutation, only one of the two mutant samples was called (Fig. 7). This can be attributed to both the suboptimal specificity of the wild-type assay and the low variant allele frequency of this particular sample. When plotting the ddPCR variant allele frequency in function of the DMAS dCq values, a very good correlation is observed, indicating that the results obtained through DMAS-qPCR reflect the genotype and variant allele frequency of the sample very well (Fig. 8).

### Implementation in primerXL

The design of DMAS-qPCR genotyping assays has been implemented in our web tool primerXL to increase the usability of the method (www.primerxl.org). Design requests can be submitted using a reference SNP identification number (dbSNP rs ID), a sequence with the mutation annotation between brackets or a chromosomal location accompanied by the WT and mutant allele(s). The mutation information – retrieved from the Ensembl server in case a rs number is submitted – is then fed into the design pipeline resulting in the generation of three primers: one common primer and two allele-specific primers. All assays undergo *in silico* specificity analysis and assessment of secondary structures in the primer annealing sites. Available Primer3^5^ options such as annealing temperature, amplicon length and others parameters are also customizable. The primerXL genotyping assay design success rate is 69.8% (624 out of 894 randomly selected SNPs using identical design settings), with 80.7% (218/270) of the failed SNPs located in repeats (according to Ensembl release 75). Design success rate can be increased by optimizing the design settings on a mutation specific basis. Currently, primerXL is only capable of designing assays for single nucleotide substitutions, but the same principles could also be applied to larger substitutions, insertions and deletions. This functionality will be implemented in future versions.

## Discussion

Our study on the impact of primer-template mismatches on qPCR performance suggested novel options for design of a cost-efficient and straightforward method for SNP genotyping or mutation detection. Initial experiments indicated that an artificial mismatch in the primer sequence needs to be introduced as a single mismatch at the 3′ end of the allele-specific primer does not have sufficient discriminative power. Additional tests showed that an artificial mismatch at the fourth nucleotide from the 3′ end of the allele-specific primer has the best overall performance, independent of the type of artificial mismatch. Genotyping of twelve SNPs involved in the *TP53* pathway and 15 clinically relevant mutations on 48 neuroblastoma cell lines demonstrated high specificity, resolution and robustness of the assays. However, for rs34330 clustering of the different genotypes was less pronounced which can be attributed to the higher Cq values of this assay compared to the others. The sensitivity of the assays in the context of WT/mutant hybrid samples ranges from 0.05 to 6.25%. However, we hypothesize that a small modification to the method – such as chemically modified allele-specific primers with higher binding affinities – could help increase overall sensitivity. Through automated instruments, DMAS-qPCR could be performed in high-throughput contexts. However, for large sets of SNPs, the method is outperformed by MS-based genotyping both in the context of scalability and cost-efficiency. In comparison to other qPCR based genotyping methods such as hydrolysis probes and high-resolution melting curve analysis, DMAS-qPCR has the benefit that no probes or specialized instruments are needed. This results in a reduction of the reaction complexity and cost of the assay. Comparison of DMAS-qPCR genotype calling with TaqMan results for twelve SNPs in 48 cell lines and 2 clinically relevant mutations on 40 cell lines, showed a hundred percent calling success rate with 547 correct calls out of 576 (98.9% concordance) genotypes and 79 correct calls out of 80 genotypes (98.75% concordance), respectively. TaqMan assays resulted in approximately four percent undetermined calls, indicating a higher robustness for DMAS-qPCR genotyping assays. Possible limitations of our method include the difficulty to assess genotype status in difficult DNA regions (e.g. homologous or homopolymeric regions due to the limited assay design space) and the use of more input DNA since two parallel qPCR reactions must be performed for each SNP or mutation to be genotyped. However, the latter problem could be circumvented by reducing the DNA input amounts per reaction, as data have shown that even low DNA input amounts result in acceptable analytical sensitivities. An alternative but yet untested approach would entail the addition of a GC tail to one off the AS primers of a DMAS-qPCR assay, enabling genotype calling using a single reaction by means of melting curves instead of (d)Cq values. Also, to enable DMAS-qPCR based high-throughput genotype calling a classification or clustering method would be preferred instead of the cut-off based calling performed in this study. Finally, by utilizing synthetic templates, this study introduced the idea of using these molecules as positive controls for genotyping reactions. Together with the user-friendly assay design in primerXL, our fast, simple and cost-effective qPCR based genotyping technique, combined with a new visualization and analysis method under the form of a difference plot, is a powerful alternative to the commonly used genotyping methods.

## Supplementary information

Supplementary data
