# Supplementary material for: Cost-effective and robust genotyping using double-mismatch allele-specific quantitative PCR
Source: Sci Rep. 2019 Feb 15;9:2150. doi: 10.1038/s41598-019-38581-z (PMC6377641; doi:10.1038/s41598-019-38581-z)
Supplement: Supplementary file 1 — Supplementary data [file 41598_2019_38581_MOESM1_ESM.pdf]

Cost-effective and robust genotyping using double-mismatch allele-specific quantitative PCR

Lefever Steve, Rihani Ali, Van der Meulen Joni, Pattyn Filip, Van Maerken Tom, Van Dorpe Jo, Hellemans Jan, Vandesompele Jo

Supplemental Figure 1

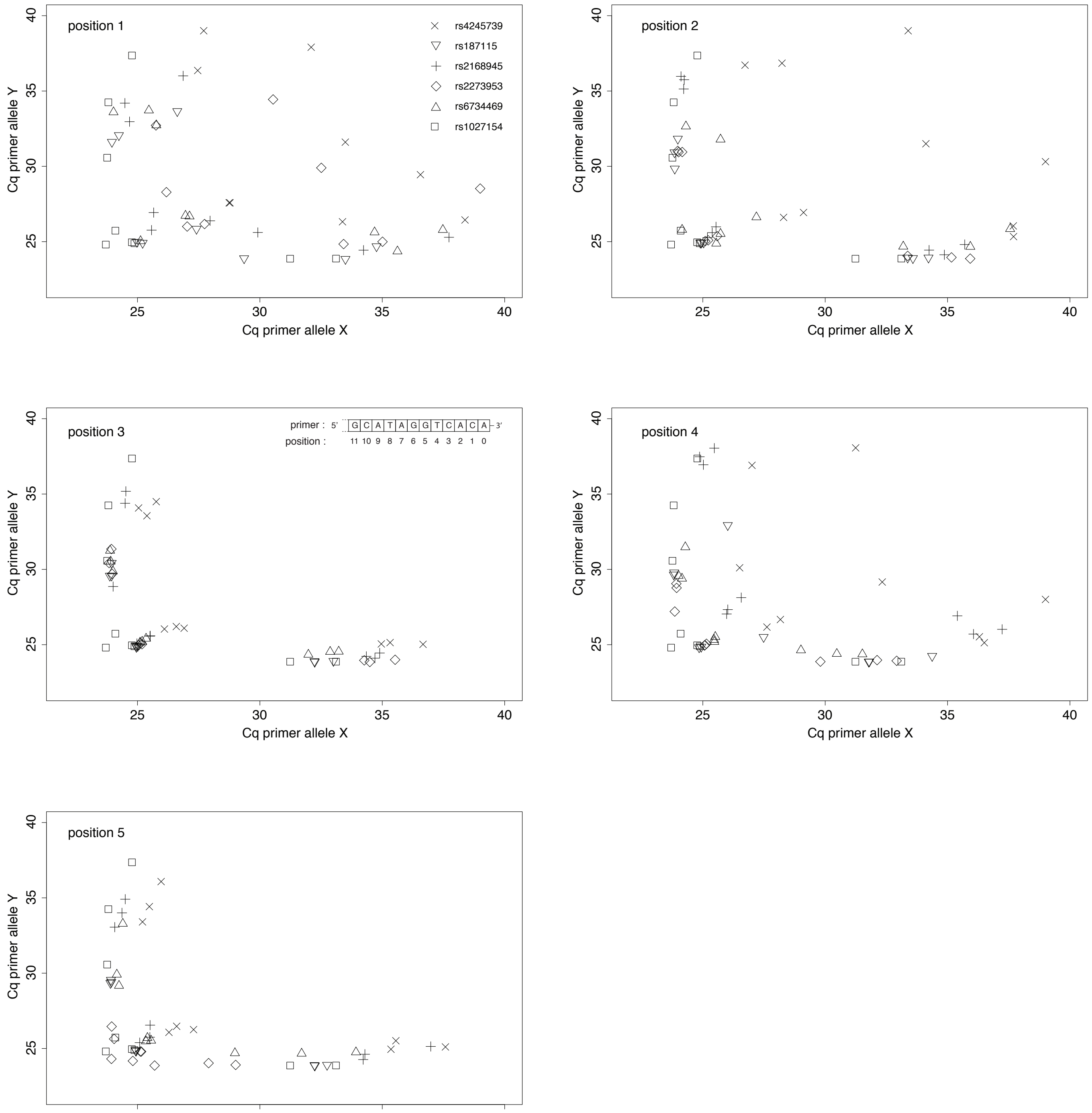

Legend : Two dimensional plot created by plotting the mean Cq values of the two reactions on opposite axes (see Figure 1) – In these plots samples with a heterozygous genotype, having two low Cq values, are grouped in the lower left corner of the plot while homozygous samples appear either in the upper left or lower right corner. The plots above display the results of the 6 SNP assays harboring an artificial mismatch on positions 1 to 5, to identify the optimal artificial mismatch position.

Supplemental Table 1

SNP	dCq			ddCq	
	A/B	A/A	B/B	AA-AB	BB-AB
rs6734469	1.89 (± 0.00)	6.22 (± 4.83)	-12.83 (± 0.00)	4.33	14.72
rs2273953	0.68 (± 0.67)	1.62 (± 1.68)	-3.61 (± 0.74)	0.95	4.29
rs187115	0.30 (±0.20)	0.10 (±0.05)	-0.19 (± 0.00)	0.21	0.49
rs4245739	1.12 (± 0.00)	1.40 (± 0.00)	-11.53 (± 1.58)	0.28	12.65
rs1027154	0.40 (± 0.00)	10.73 (± 1.37)	-2.63 (± 0.00)	10.33	3.03
rs2168945	3.79 (± 0.64)	6.14 (± 0.00)	-1.39 (± 2.16)	2.35	5.18

Legend : Mean Cq difference (dCq +/- standard deviation) between the perfect and mismatch reactions – without artificial mismatch – for six SNPs tested on homozygous (A/A and B/B) and heterozygous (A/B) samples, and ddCq values – calculated as the absolute difference between the mean dCq value of the heterozygous samples and the dCq value of homozygous samples – representing the degree in discriminating power.

Supplemental Table 2

	rs4245739		
cell line name	DMAS-qPCR	TaqMan - genotyper	TaqMan - manual
NBL-S	A/C	A/C	A/C
STA-NB-12	A/A	A/A	A/A
NB-1	A/A	A/A	A/A
SK-N-FI	A/C	A/C	A/C
CLB-GA	C/C	C/C	C/C
SKNBE(2c)	A/A	A/A	A/A
NLF	C/C	C/C	C/C
IMR-32	A/C	A/C	A/C
SK-N-AS	A/A	A/A	A/A
CHP-134	A/C	no calling possible	A/C
CHP-901	A/A	A/A	A/A
N-206	A/A	A/A	A/A
SJNB-8	A/C	A/C	A/C
SJNB-6	A/A	A/A	A/A
GIMEN	A/C	A/C	A/C
SH-SY5Y	A/C	A/C	A/C
SHEP	A/C	A/C	A/C
NGP	A/C	A/C	A/C
SKNBE	A/A	A/A	A/A
CHP-902R	A/C	A/C	A/C
SKNSH	A/C	A/C	A/C
SJNB-10	C/C	C/C	C/C
NMB	A/C	no calling possible	A/C
TR-14	A/A	A/A	A/A
SMS-KCNR	A/C	A/C	A/C
GICIN-1	A/C	A/C	A/C
STA-NB-10	A/C	A/C	A/C
SJNB-12	A/C	A/C	A/C
SMS-KAN	A/A	A/A	A/A
LAN-5	A/C	A/C	A/C
UKF-NB3	A/C	A/C	A/C
STA-NB-8	A/A	A/A	A/A
STA-NB-3	A/C	A/C	A/C
SKMYC2	A/C	A/C	A/C
STA-NB-9	A/C	A/C	A/C
SJNB1 (1) 30/04/1999	A/A	A/A	A/A
LAN-2	A/A	A/A	A/A
NB-13	A/A	A/A	A/A
ACN	A/A	A/A	A/A
Kelly	A/A	A/A	A/A
NB-5	A/A	A/A	A/A
UHG-NP	A/A	A/A	A/A
LA-N-6	A/C	A/C	A/C
CHLA-90	A/A	A/A	A/A
LA-N-6	A/C	A/C	A/C
LA-N-1	A/A	A/A	A/A
SMS-KCN	A/C	A/C	A/C
UKF-NB-2	A/C	A/C	A/C

	rs2273953		
cell line name	DMAS-qPCR	TaqMan - genotyper	TaqMan - manual
NBL-S	G/G	G/G	G/G
STA-NB-12	A/A	A/A	A/A
NB-1	G/G	G/G	G/G
SK-N-FI	G/G	G/G	G/G
CLB-GA	G/G	G/G	G/G
SKNBE(2c)	G/G	G/G	G/G
NLF	G/G	G/G	G/G
IMR-32	A/A	A/A	A/A
SK-N-AS	G/G	G/G	G/G
CHP-134	G/G	G/G	G/G
CHP-901	G/G	G/G	G/G
N-206	G/G	G/G	G/G
SJNB-8	G/G	G/G	G/G
SJNB-6	G/G	G/G	G/G
GIMEN	G/G	G/G	G/G
SH-SY5Y	A/G	no calling possible	A/G
SHEP	A/G	no calling possible	A/G
NGP	A/G	G/G	G/G
SKNBE	G/G	G/G	G/G
CHP-902R	G/G	G/G	G/G
SKNSH	A/G	A/G	A/G
SJNB-10	G/G	G/G	G/G
NMB	G/G	G/G	G/G
TR-14	G/G	G/G	G/G
SMS-KCNR	A/A	A/A	A/A
GICIN-1	G/G	G/G	G/G
STA-NB-10	G/G	G/G	G/G
SJNB-12	G/G	G/G	G/G
SMS-KAN	G/G	G/G	G/G
LAN-5	A/A	A/A	A/A
UKF-NB3	A/G	no calling possible	no calling possible
STA-NB-8	G/G	G/G	G/G
STA-NB-3	G/G	G/G	G/G
SKMYC2	A/G	A/G	A/G
STA-NB-9	G/G	G/G	G/G
SJNB1 (1) 30/04/1999	G/G	G/G	G/G
LAN-2	G/G	G/G	G/G
NB-13	G/G	G/G	G/G
ACN	G/G	G/G	G/G
Kelly	G/G	G/G	G/G
NB-5	G/G	G/G	G/G
UHG-NP	G/G	G/G	G/G
LA-N-6	A/A	A/A	A/A
CHLA-90	G/G	G/G	G/G
LA-N-6	A/A	A/A	A/A
LA-N-1	G/G	G/G	G/G
SMS-KCN	A/A	A/A	A/A
UKF-NB-2	G/G	G/G	G/G

	rs2168945		
cell line name	DMAS-qPCR	TaqMan - genotyper	TaqMan - manual
NBL-S	C/T	C/T	C/T
STA-NB-12	T/T	T/T	T/T
NB-1	C/T	C/T	C/T
SK-N-FI	T/T	T/T	T/T
CLB-GA	C/C	C/C	C/C
SKNBE(2c)	T/T	T/T	T/T
NLF	C/C	C/C	C/C
IMR-32	C/T	C/T	C/T
SK-N-AS	C/C	C/C	C/C
CHP-134	T/T	T/T	T/T
CHP-901	C/T	C/T	C/T
N-206	C/T	C/T	C/T
SJNB-8	T/T	T/T	T/T
SJNB-6	T/T	T/T	T/T
GIMEN	C/T	no calling possible	C/T
SH-SY5Y	C/T	C/T	C/T
SHEP	C/T	C/T	C/T
NGP	C/C	C/C	C/C
SKNBE	T/T	T/T	T/T
CHP-902R	C/T	C/T	C/T
SKNSH	C/T	C/T	C/T
SJNB-10	C/T	C/T	C/T
NMB	C/T	C/T	C/T
TR-14	T/T	T/T	T/T
SMS-KCNR	C/T	C/T	C/T
GICIN-1	T/T	T/T	T/T
STA-NB-10	T/T	T/T	T/T
SJNB-12	C/C	C/C	C/C
SMS-KAN	C/T	C/T	C/T
LAN-5	C/C	C/C	C/C
UKF-NB3	C/T	C/T	C/T
STA-NB-8	T/T	T/T	T/T
STA-NB-3	T/T	T/T	T/T
SKMYC2	C/T	C/T	C/T
STA-NB-9	C/T	C/T	C/T
SJNB1 (1) 30/04/1999	C/T	no calling possible	C/T
LAN-2	C/T	C/T	C/T
NB-13	C/T	C/T	C/T
ACN	C/C	C/C	C/C
Kelly	C/T	C/T	C/T
NB-5	C/T	C/T	C/T
UHG-NP	C/T	C/T	C/T
LA-N-6	T/T	no calling possible	T/T
CHLA-90	C/T	C/T	C/T
LA-N-6	T/T	T/T	T/T
LA-N-1	T/T	T/T	T/T
SMS-KCN	C/T	C/T	C/T
UKF-NB-2	C/T	C/T	C/T

cell line name	rs187115		
	DMAS-qPCR	TaqMan - genotyper	TaqMan - manual
NBL-S	A/C	A/C	A/C
STA-NB-12	A/A	no calling possible	no calling possible
NB-1	A/A	A/A	A/A
SK-N-FI	A/C	A/C	A/C
CLB-GA	A/A	A/A	A/A
SKNBE(2c)	A/A	A/A	A/A
NLF	C/C	C/C	C/C
IMR-32	C/C	C/C	C/C
SK-N-AS	A/A	A/A	A/A
CHP-134	A/C	A/C	A/C
CHP-901	A/A	A/A	A/A
N-206	A/C	no calling possible	no calling possible
SJNB-8	A/C	A/C	A/C
SJNB-6	A/C	A/C	A/C
GIMEN	A/C	A/C	A/C
SH-SY5Y	C/C	C/C	C/C
SHEP	C/C	C/C	C/C
NGP	A/C	A/C	A/C
SKNBE	A/A	A/A	A/A
CHP-902R	A/C	A/C	A/C
SKNSH	C/C	C/C	C/C
SJNB-10	A/C	A/C	A/C
NMB	A/A	A/A	A/A
TR-14	A/A	A/A	A/A
SMS-KCNR	A/A	A/A	A/A
GICIN-1	C/C	C/C	C/C
STA-NB-10	C/C	C/C	C/C
SJNB-12	A/A	A/A	A/A
SMS-KAN	A/A	A/A	A/A
LAN-5	A/C	A/C	A/C
UKF-NB3	A/A	A/A	A/A
STA-NB-8	A/A	A/A	A/A
STA-NB-3	A/C	no calling possible	no calling possible
SKMYC2	C/C	C/C	C/C
STA-NB-9	A/A	A/A	A/A
SJNB1 (1) 30/04/1999	A/C	A/C	A/C
LAN-2	A/A	no calling possible	no calling possible
NB-13	C/C	A/A	A/A
ACN	C/C	C/C	C/C
Kelly	A/C	A/C	A/C
NB-5	A/C	A/C	A/C
UHG-NP	A/A	A/A	A/A
LA-N-6	A/C	A/C	A/C
CHLA-90	A/A	A/A	A/A
LA-N-6	A/C	A/C	A/C
LA-N-1	C/C	C/C	C/C
SMS-KCN	A/A	A/A	A/A
UKF-NB-2	A/C	no calling possible	A/C

	rs1027154		
cell line name	DMAS-qPCR	TaqMan - genotyper	TaqMan - manual
NBL-S	G/C	G/C	G/C
STA-NB-12	G/C	G/C	G/C
NB-1	G/G	G/G	G/G
SK-N-FI	G/G	G/G	G/G
CLB-GA	G/G	G/G	G/G
SKNBE(2c)	G/G	G/G	G/G
NLF	G/C	G/C	G/C
IMR-32	G/G	G/G	G/G
SK-N-AS	G/G	G/G	G/G
CHP-134	G/G	G/G	G/G
CHP-901	G/G	G/G	G/G
N-206	G/C	G/C	G/C
SJNB-8	G/G	G/G	G/G
SJNB-6	G/C	G/C	G/C
GIMEN	G/G	G/G	G/G
SH-SY5Y	G/C	G/C	G/C
SHEP	G/C	G/C	G/C
NGP	G/G	G/G	G/G
SKNBE	G/G	G/G	G/G
CHP-902R	G/G	G/G	G/G
SKNSH	G/C	G/C	G/C
SJNB-10	G/G	G/G	G/G
NMB	G/G	G/G	G/G
TR-14	G/G	G/G	G/G
SMS-KCNR	G/G	G/G	G/G
GICIN-1	G/G	G/G	G/G
STA-NB-10	G/G	G/G	G/G
SJNB-12	G/G	G/G	G/G
SMS-KAN	G/C	G/C	G/C
LAN-5	G/C	G/C	G/C
UKF-NB3	G/G	G/G	G/G
STA-NB-8	G/G	G/G	G/G
STA-NB-3	G/G	G/G	G/G
SKMYC2	G/C	G/C	G/C
STA-NB-9	G/G	G/G	G/G
SJNB1 (1) 30/04/1999	G/G	G/G	G/G
LAN-2	G/C	G/C	G/C
NB-13	G/G	G/G	G/G
ACN	G/G	G/G	G/G
Kelly	G/C	G/C	G/C
NB-5	G/G	G/G	G/G
UHG-NP	G/G	G/G	G/G
LA-N-6	G/G	G/G	G/G
CHLA-90	G/C	G/C	G/C
LA-N-6	G/G	G/G	G/G
LA-N-1	G/C	G/C	G/C
SMS-KCN	G/G	G/G	G/G
UKF-NB-2	G/G	G/G	G/G

	rs6734469		
cell line name	DMAS-qPCR	TaqMan - genotyper	TaqMan - manual
NBL-S	A/G	no calling possible	A/G
STA-NB-12	G/G	G/G	G/G
NB-1	G/G	G/G	G/G
SK-N-FI	A/A	no calling possible	no calling possible
CLB-GA	A/G	A/G	A/G
SKNBE(2c)	G/G	G/G	G/G
NLF	A/G	A/G	A/G
IMR-32	A/A	A/A	A/A
SK-N-AS	A/G	A/G	A/G
CHP-134	A/G	A/G	A/G
CHP-901	A/G	no calling possible	A/G
N-206	A/A	A/A	A/A
SJNB-8	G/G	G/G	G/G
SJNB-6	A/G	A/G	A/G
GIMEN	A/A	A/A	A/A
SH-SY5Y	G/G	G/G	G/G
SHEP	G/G	G/G	G/G
NGP	A/G	no calling possible	A/G
SKNBE	G/G	G/G	G/G
CHP-902R	A/G	A/G	A/G
SKNSH	G/G	G/G	G/G
SJNB-10	A/G	A/G	A/G
NMB	A/A	A/A	A/A
TR-14	A/G	no calling possible	A/G
SMS-KCNR	A/G	A/G	A/G
GICIN-1	A/A	A/A	A/A
STA-NB-10	A/A	A/A	A/A
SJNB-12	G/G	G/G	G/G
SMS-KAN	G/G	G/G	G/G
LAN-5	A/G	A/G	A/G
UKF-NB3	A/A	A/A	A/A
STA-NB-8	G/G	G/G	G/G
STA-NB-3	G/G	G/G	G/G
SKMYC2	G/G	G/G	G/G
STA-NB-9	A/A	A/A	A/A
SJNB1 (1) 30/04/1999	A/G	A/G	A/G
LAN-2	A/A	A/A	A/A
NB-13	A/A	A/A	A/A
ACN	A/A	A/A	A/A
Kelly	A/A	A/A	A/A
NB-5	A/A	A/A	A/A
UHG-NP	A/G	no calling possible	A/G
LA-N-6	A/G	A/G	A/G
CHLA-90	A/G	A/G	A/G
LA-N-6	A/G	A/G	A/G
LA-N-1	A/G	A/G	A/G
SMS-KCN	A/G	A/G	A/G
UKF-NB-2	A/G	A/G	A/G

cell line name	rs2069347		
	DMAS-qPCR	TaqMan - genotyper	TaqMan - manual
NBL-S	C/C	C/C	C/C
STA-NB-12	C/C	C/C	C/C
NB-1	T/T	T/T	T/T
SK-N-FI	C/T	C/T	C/T
CLB-GA	C/T	C/T	C/T
SKNBE(2c)	C/C	C/C	C/C
NLF	C/C	C/C	C/C
IMR-32	C/C	C/C	C/C
SK-N-AS	C/C	C/C	C/C
CHP-134	C/T	C/T	C/T
CHP-901	C/T	C/T	C/T
N-206	T/T	T/T	T/T
SJNB-8	C/C	C/C	C/C
SJNB-6	C/C	C/C	C/C
GIMEN	C/C	C/C	C/C
SH-SY5Y	C/T	C/T	C/T
SHEP	C/T	C/T	C/T
NGP	T/T	T/T	T/T
SKNBE	C/C	C/C	C/C
CHP-902R	C/C	C/C	C/C
SKNSH	C/T	C/T	C/T
SJNB-10	C/C	C/C	C/C
NMB	T/T	T/T	T/T
TR-14	C/T	C/T	C/T
SMS-KCNR	C/C	C/C	C/C
GICIN-1	C/C	C/C	C/C
STA-NB-10	C/C	C/C	C/C
SJNB-12	C/T	C/T	C/T
SMS-KAN	C/T	C/T	C/T
LAN-5	C/C	C/C	C/C
UKF-NB3	C/T	no calling possible	C/T
STA-NB-8	C/T	C/T	C/T
STA-NB-3	C/C	C/C	C/C
SKMYC2	C/T	C/T	C/T
STA-NB-9	C/C	C/C	C/C
SJNB1 (1) 30/04/1999	T/T	T/T	T/T
LAN-2	C/C	C/C	C/C
NB-13	C/T	C/T	C/T
ACN	C/T	no calling possible	C/T
Kelly	T/T	T/T	T/T
NB-5	C/T	C/T	C/T
UHG-NP	T/T	T/T	T/T
LA-N-6	T/T	T/T	T/T
CHLA-90	C/C	C/C	C/C
LA-N-6	T/T	T/T	T/T
LA-N-1	C/T	C/T	C/T
SMS-KCN	C/C	C/C	C/C
UKF-NB-2	C/T	C/C	C/T

cell line name	rs34330		
	DMAS-qPCR	TaqMan - genotyper	TaqMan - manual
NBL-S	C/T	C/T	C/T
STA-NB-12	C/T	C/T	C/T
NB-1	T/T	T/T	T/T
SK-N-FI	C/C	C/C	C/C
CLB-GA	T/T	T/T	T/T
SKNBE(2c)	T/T	T/T	T/T
NLF	C/T	C/T	C/T
IMR-32	C/T	C/T	C/T
SK-N-AS	T/T	T/T	T/T
CHP-134	C/C	C/C	C/C
CHP-901	T/T	T/T	T/T
N-206	T/T	T/T	T/T
SJNB-8	C/T	C/T	C/T
SJNB-6	C/T	C/T	C/T
GIMEN	C/T	C/T	C/T
SH-SY5Y	C/C	C/C	C/C
SHEP	C/C	C/C	C/C
NGP	C/C	C/C	C/C
SKNBE	T/T	T/T	T/T
CHP-902R	C/C	C/C	C/C
SKNSH	C/C	C/C	C/C
SJNB-10	C/T	C/T	C/T
NMB	T/T	T/T	T/T
TR-14	T/T	T/T	T/T
SMS-KCNR	C/C	C/C	C/C
GICIN-1	C/C	C/C	C/C
STA-NB-10	C/T	C/T	C/T
SJNB-12	C/T	C/T	C/T
SMS-KAN	C/T	C/T	C/T
LAN-5	C/T	C/T	C/T
UKF-NB3	C/C	C/C	C/C
STA-NB-8	T/T	T/T	T/T
STA-NB-3	C/T	no calling possible	C/T
SKMYC2	C/C	C/C	C/C
STA-NB-9	C/T	C/T	C/T
SJNB1 (1) 30/04/1999	C/T	C/T	C/T
LAN-2	C/C	C/C	C/C
NB-13	T/T	T/T	T/T
ACN	C/C	C/C	C/C
Kelly	T/T	T/T	T/T
NB-5	T/T	T/T	T/T
UHG-NP	C/C	C/C	C/C
LA-N-6	T/T	T/T	T/T
CHLA-90	C/C	C/C	C/C
LA-N-6	T/T	T/T	T/T
LA-N-1	C/T	C/T	C/T
SMS-KCN	C/C	C/C	C/C
UKF-NB-2	C/T	T/T	C/T

cell line name	rs2426127 DMAS-qPCR	TaqMan - genotyper	TaqMan - manual
NBL-S	C/C	C/C	C/C
STA-NB-12	C/C	C/C	C/C
NB-1	T/T	T/T	T/T
SK-N-FI	C/T	C/T	C/T
CLB-GA	C/C	C/C	C/C
SKNBE(2c)	C/C	C/C	C/C
NLF	C/T	C/T	C/T
IMR-32	C/C	C/C	C/C
SK-N-AS	C/T	C/T	C/T
CHP-134	C/T	C/T	C/T
CHP-901	T/T	T/T	T/T
N-206	C/T	C/T	C/T
SJNB-8	C/T	C/T	C/T
SJNB-6	C/T	C/T	C/T
GIMEN	C/C	C/C	C/C
SH-SY5Y	T/T	T/T	T/T
SHEP	T/T	T/T	T/T
NGP	C/C	C/C	C/C
SKNBE	C/C	C/C	C/C
CHP-902R	C/C	C/C	C/C
SKNSH	T/T	T/T	T/T
SJNB-10	C/C	C/C	C/C
NMB	C/T	C/T	C/T
TR-14	C/T	C/T	C/T
SMS-KCNR	C/C	C/C	C/C
GICIN-1	C/C	C/C	C/C
STA-NB-10	T/T	T/T	T/T
SJNB-12	C/T	C/T	C/T
SMS-KAN	C/T	C/T	C/T
LAN-5	C/T	C/T	C/T
UKF-NB3	C/C	C/C	C/C
STA-NB-8	C/T	C/T	C/T
STA-NB-3	T/T	T/T	T/T
SKMYC2	T/T	T/T	T/T
STA-NB-9	C/C	C/C	C/C
SJNB1 (1) 30/04/1999	C/C	C/C	C/C
LAN-2	C/C	C/C	C/C
NB-13	C/C	C/C	C/C
ACN	C/C	C/C	C/C
Kelly	C/T	C/T	C/T
NB-5	C/C	C/C	C/C
UHG-NP	C/C	C/C	C/C
LA-N-6	C/C	C/C	C/C
CHLA-90	C/C	C/C	C/C
LA-N-6	C/C	C/C	C/C
LA-N-1	C/T	C/T	C/T
SMS-KCN	C/C	C/C	C/C
UKF-NB-2	C/C	C/C	C/C

cell line name	rs1800054 DMAS-qPCR	TaqMan - genotyper	TaqMan - manual
NBL-S	C/C	C/C	C/C
STA-NB-12	C/C	C/C	C/C
NB-1	C/C	C/C	C/C
SK-N-FI	C/C	C/C	C/C
CLB-GA	C/C	C/C	C/C
SKNBE(2c)	C/C	C/C	C/C
NLF	C/C	C/C	C/C
IMR-32	C/C	C/C	C/C
SK-N-AS	C/C	C/C	C/C
CHP-134	C/C	C/C	C/C
CHP-901	C/C	C/C	C/C
N-206	C/C	C/C	C/C
SJNB-8	C/C	C/C	C/C
SJNB-6	C/C	C/C	C/C
GIMEN	C/C	C/C	C/C
SH-SY5Y	C/C	C/C	C/C
SHEP	C/C	C/C	C/C
NGP	C/C	C/C	C/C
SKNBE	C/C	C/C	C/C
CHP-902R	C/C	C/C	C/C
SKNSH	C/C	C/C	C/C
SJNB-10	C/C	C/C	C/C
NMB	C/C	C/C	C/C
TR-14	C/C	C/C	C/C
SMS-KCNR	C/C	C/C	C/C
GICIN-1	C/C	C/C	C/C
STA-NB-10	C/C	C/C	C/C
SJNB-12	C/C	C/C	C/C
SMS-KAN	C/C	C/C	C/C
LAN-5	C/C	C/C	C/C
UKF-NB3	C/C	C/C	C/C
STA-NB-8	C/C	C/C	C/C
STA-NB-3	C/C	C/C	C/C
SKMYC2	C/C	C/C	C/C
STA-NB-9	C/G	C/G	C/G
SJNB1 (1) 30/04/1999	C/C	C/C	C/C
LAN-2	C/C	C/C	C/C
NB-13	C/C	C/C	C/C
ACN	C/C	C/C	C/C
Kelly	C/C	C/C	C/C
NB-5	C/C	C/C	C/C
UHG-NP	C/C	C/C	C/C
LA-N-6	C/C	C/C	C/C
CHLA-90	C/C	C/C	C/C
LA-N-6	C/C	C/C	C/C
LA-N-1	C/C	C/C	C/C
SMS-KCN	C/C	C/C	C/C
UKF-NB-2	C/C	C/C	C/C

	rs1045485		
cell line name	DMAS-qPCR	TaqMan - genotyper	TaqMan - manual
NBL-S	G/G	G/G	G/G
STA-NB-12	G/G	G/G	G/G
NB-1	G/G	G/G	G/G
SK-N-FI	C/G	C/G	C/G
CLB-GA	C/G	C/G	C/G
SKNBE(2c)	G/G	G/G	G/G
NLF	C/G	C/G	C/G
IMR-32	G/G	G/G	G/G
SK-N-AS	G/G	G/G	G/G
CHP-134	G/G	G/G	G/G
CHP-901	G/G	G/G	G/G
N-206	G/G	G/G	G/G
SJNB-8	G/G	G/G	G/G
SJNB-6	G/G	G/G	G/G
GIMEN	G/G	G/G	G/G
SH-SY5Y	G/G	G/G	G/G
SHEP	G/G	G/G	G/G
NGP	G/G	G/G	G/G
SKNBE	G/G	G/G	G/G
CHP-902R	G/G	G/G	G/G
SKNSH	G/G	G/G	G/G
SJNB-10	G/G	G/G	G/G
NMB	C/G	C/G	C/G
TR-14	C/G	C/G	C/G
SMS-KCNR	C/G	C/G	C/G
GICIN-1	G/G	G/G	G/G
STA-NB-10	C/C	C/C	C/C
SJNB-12	G/G	G/G	G/G
SMS-KAN	G/G	G/G	G/G
LAN-5	G/G	G/G	G/G
UKF-NB3	C/G	C/G	C/G
STA-NB-8	G/G	G/G	G/G
STA-NB-3	G/G	G/G	G/G
SKMYC2	G/G	G/G	G/G
STA-NB-9	G/G	G/G	G/G
SJNB1 (1) 30/04/1999	G/G	G/G	G/G
LAN-2	G/G	G/G	G/G
NB-13	G/G	G/G	G/G
ACN	G/G	G/G	G/G
Kelly	G/G	G/G	G/G
NB-5	G/G	G/G	G/G
UHG-NP	G/G	G/G	G/G
LA-N-6	G/G	G/G	G/G
CHLA-90	G/G	G/G	G/G
LA-N-6	G/G	G/G	G/G
LA-N-1	G/G	G/G	G/G
SMS-KCN	C/G	C/G	C/G
UKF-NB-2	C/G	G/G	no calling possible

cell line name	rs319227 DMAS-qPCR	TaqMan - genotyper	TaqMan - manual
NBL-S	A/C	A/C	A/C
STA-NB-12	C/C	C/C	C/C
NB-1	A/C	A/C	A/C
SK-N-FI	C/C	C/C	C/C
CLB-GA	A/C	A/C	A/C
SKNBE(2c)	A/C	A/C	A/C
NLF	A/A	A/A	A/A
IMR-32	A/C	A/C	A/C
SK-N-AS	A/A	A/A	A/A
CHP-134	C/C	C/C	C/C
CHP-901	C/C	C/C	C/C
N-206	A/A	A/A	A/A
SJNB-8	A/A	A/A	A/A
SJNB-6	A/C	A/C	A/C
GIMEN	A/C	A/C	A/C
SH-SY5Y	A/C	A/C	A/C
SHEP	A/C	A/C	A/C
NGP	C/C	C/C	C/C
SKNBE	A/C	A/C	A/C
CHP-902R	A/C	A/C	A/C
SKNSH	A/C	A/C	A/C
SJNB-10	A/C	A/C	A/C
NMB	C/C	C/C	C/C
TR-14	A/C	no calling possible	A/C
SMS-KCNR	A/A	A/A	A/A
GICIN-1	A/C	A/C	A/C
STA-NB-10	A/A	A/A	A/A
SJNB-12	A/A	A/A	A/A
SMS-KAN	A/C	A/C	A/C
LAN-5	A/A	A/A	A/A
UKF-NB3	A/A	A/A	A/A
STA-NB-8	C/C	C/C	C/C
STA-NB-3	A/A	A/A	A/A
SKMYC2	A/C	A/C	A/C
STA-NB-9	A/C	A/C	A/C
SJNB1 (1) 30/04/1999	A/A	A/A	A/A
LAN-2	A/A	A/A	A/A
NB-13	A/C	A/C	A/C
ACN	C/C	C/C	C/C
Kelly	C/C	A/A	A/A
NB-5	C/C	C/C	C/C
UHG-NP	A/C	A/C	A/C
LA-N-6	C/C	C/C	C/C
CHLA-90	A/C	A/C	A/C
LA-N-6	C/C	C/C	C/C
LA-N-1	A/C	A/C	A/C
SMS-KCN	A/A	A/A	A/A
UKF-NB-2	A/C	A/C	A/C

Legend : DMAS-qPCR and TaqMan genotype calls for 12 TP53 related SNPs in 48 neuroblastoma cell lines

Supplemental Table 3

cell line name	ALK F1174L G>T DMAS-qPCR	ALK F1174L G>T TaqMan	included in comparison
ACN	C/C	no data available	no
CHLA-90	C/C	no data available	no
CHP-134	C/C	wt	yes
CHP-901	C/C	wt	yes
CHP-902R	C/C	wt	yes
CLB-GA	C/C	wt	yes
gDNA	C/C	wt	no
GICIN-1	C/C	wt	yes
GIMEN	C/C	wt	yes
IMR-32	C/C	wt	yes
Kelly	A/C	no data available	no
LA-N-1	A/C	F1174L	yes
LA-N-6 (T10-282)	C/C	wt	yes
LA-N-6 (T10-295)	C/C	wt	yes
LAN-2	C/C	wt	yes
LAN-5	C/C	wt	yes
N-206	A/C	F1174L	yes
NB-1	C/C	wt	yes
NB-13	C/C	wt	yes
NB-5	C/C	wt	yes
NBL-S	C/C	wt	yes
NGP	C/C	wt	yes
NLF	C/C	wt	yes
NMB	C/C	wt	yes
SH-SY5Y	A/C	F1174L	yes
SHEP	A/C	F1174L	yes
SJNB-10	C/C	wt	yes
SJNB-12	C/C	no data available	no
SJNB-6	C/C	wt	yes
SJNB-8	C/C	wt	yes
SJNB1 (1) - 30/04/1999	C/C	no calling possible	no
SK-N-AS	C/C	wt	yes
SK-NF-I	C/C	wt	yes
SKMYC2	A/C	no data available	no
SKNBE	C/C	wt	yes
SKNBE(2c)	C/C	wt	yes
SKNSH	A/C	F1174L	yes
SMS-KAN	C/C	wt	yes
SMS-KCN	C/C	no data available	no
SMS-KCNR	A/C	F1174L	yes
STA-NB-10	C/C	wt	yes
STA-NB-12	C/C	wt	yes
STA-NB-3	C/C	wt	yes
STA-NB-8	C/C	F1174L	yes
STA-NB-9	C/C	wt	yes
TR-14	C/C	wt	yes
UHG-NP	C/C	wt	yes
UKF-NB3	C/C	wt	yes

cell line name	ALK R1275Q DMAS-qPCR	TaqMan	included in comparison
ACN	C/C	no data available	no
CHLA-90	C/C	no data available	no
CHP-134	C/C	wt	yes
CHP-901	C/C	wt	yes
CHP-902R	C/C	wt	yes
CLB-GA	C/T	R1275Q	yes
gDNA	C/C	wt	no
GICIN-1	C/C	wt	yes
GIMEN	C/C	wt	yes
IMR-32	C/C	wt	yes
Kelly	C/C	no data available	no
LA-N-1	C/C	wt	yes
LA-N-6 (T10-282)	C/C	wt	yes
LA-N-6 (T10-295)	C/C	wt	yes
LAN-2	C/C	wt	yes
LAN-5	C/T	R1275Q	yes
N-206	C/C	wt	yes
NB-1	C/C	wt	yes
NB-13	C/C	wt	yes
NB-5	C/C	wt	yes
NBL-S	C/C	wt	yes
NGP	C/C	wt	yes
NLF	C/C	wt	yes
NMB	C/C	wt	yes
SH-SY5Y	C/C	wt	yes
SHEP	C/C	wt	yes
SJNB-10	C/C	wt	yes
SJNB-12	C/C	no data available	no
SJNB-6	C/C	wt	yes
SJNB-8	C/C	wt	yes
SJNB1 (1) - 30/04/1999	C/C	no calling possible	no
SK-N-AS	C/C	wt	yes
SK-NF-I	C/C	wt	yes
SKMYC2	C/C	no data available	no
SKNBE	C/C	wt	yes
SKNBE(2c)	C/C	wt	yes
SKNSH	C/C	wt	yes
SMS-KAN	C/C	wt	yes
SMS-KCN	no calling possible	no data available	no
SMS-KCNR	C/C	wt	yes
STA-NB-10	C/C	wt	yes
STA-NB-12	C/C	wt	yes
STA-NB-3	C/C	wt	yes
STA-NB-8	C/C	wt	yes
STA-NB-9	C/C	wt	yes
TR-14	C/C	wt	yes
UHG-NP	C/C	wt	yes
UKF-NB3	C/T	R1275Q	yes

Legend : DMAS-qPCR and TaqMan genotype calls for ALK F1174 G>T and R1275Q mutations in 48 neuroblastoma cell lines
